# Dataset on neonatal and maternal factors influencing neurodevelopmental outcomes in preterm infants: A study focused on the healthcare context of Mashhad, Iran

**DOI:** 10.1016/j.dib.2024.110058

**Published:** 2024-01-11

**Authors:** Azadeh Darabi, Raheleh Faramarzi, Hassan Boskabadi, Gholamali Maamouri, Reyhane Rezvani

**Affiliations:** Department of Paediatrics, Faculty of Medicine, Mashhad University of Medical Sciences, Mashhad, Iran

**Keywords:** Bayley scales of infant and toddler development, Neonatal intensive care unit (NICU), Intrauterine growth restriction (IUGR), Bronchopulmonary dysplasia (BPD), Gestational age, Pneumothorax

## Abstract

This dataset offers an insight into the neurodevelopmental trajectories of preterm infants, encapsulating a wide array of neonatal and maternal factors. The data variables include demographic details alongside a detailed account of maternal health during pregnancy, encompassing aspects and other complications. Furthermore, the dataset documents neonatal health conditions. It also records critical indicators of neonatal health. The dataset is enriched with data on medical interventions and hospitalization details. It also contains information on the mother's drug usage during pregnancy and sonography results. A significant portion of the dataset is dedicated to the developmental assessment of the infants, utilizing the Bayley Scales to evaluate various domains such as cognitive, language, perceptual, fine motor, and coarse motor skills. The data are categorized to denote normal and abnormal outcomes in these domains, providing a detailed view of the developmental progress of the infants.

The reuse potential of this dataset is substantial, serving as a rich resource for researchers and clinicians aiming to delve deeper into the multifaceted influences on preterm infant development. It can significantly contribute to the formulation of early intervention strategies, fostering a better understanding and enhancement of developmental outcomes in preterm infants.

Specifications Table

**Table:** 

Subject	Perinatology, Paediatrics and Child Health
Specific subject area	Neonatal healthcare and developmental outcomes.
Data format	Raw
Type of data	.sav file (dataset with labels) .xlsx (dataset with labels) .pdf (code book)
Data collection	Data were collected retrospectively from the medical records of preterm infants admitted to the NICU at Ghaem Hospital, Mashhad, Iran between 2016 and 2020. The developmental assessments performed by using the Bayley Scales of Infant and Toddler Development. Statistical analyses were conducted using SPSS V.26 and R software.
Data source location	The data were primarily sourced from the medical records of preterm infants admitted to the Neonatal Intensive Care Unit (NICU) at Ghaem Hospital, situated in Mashhad, Iran.
Data accessibility	Repository name: Mendeley Data https://data.mendeley.com/datasets/h464gsf77t/2
Related research article	Faramarzi R, Darabi A, Emadzadeh M, Maamouri G, Rezvani R. Predicting neurodevelopmental outcomes in preterm infants: A comprehensive evaluation of neonatal and maternal risk factors. *Early Hum Dev*. 2023;184:105834. doi:10.1016/j.earlhumdev.2023.105834[1]

## 1 Value of the Data

• This dataset is a vital resource for researchers investigating the complex factors affecting preterm infants' developmental outcomes.
• The data stands as a facilitative tool for meta-analyses and systematic reviews, aiding researchers in synthesizing existing findings and pinpointing gaps in current knowledge, steering the direction for future focused studies.

## 2 Background

The primary motivation for compiling this dataset was to explore the neurodevelopmental outcomes of preterm infants, with a particular focus on the healthcare context of Mashhad, Iran. This endeavor was rooted in a methodological framework that leverages the Bayley Scales of Infant and Toddler Development to assess a range of neonatal and maternal factors.

Our approach was driven by the theoretical understanding that a multitude of factors, including maternal health during pregnancy and neonatal conditions, play a critical role in shaping the developmental trajectories of preterm infants. The dataset, therefore, encompasses detailed demographic information, maternal health data during pregnancy, and neonatal health conditions, among other variables.

This data article complements the original research by providing a granular, data-driven view of the factors influencing neurodevelopmental outcomes in preterm infants. It serves as a resource for researchers and clinicians, offering a multidimensional perspective that underpins and extends the findings of the published research. By presenting the data in its raw, uninterpreted form, this article allows for diverse, independent analyses, thereby adding significant value to the existing research landscape.

## 3 Data Description

The dataset contains information on neonatal and maternal factors affecting the neurodevelopmental outcomes of preterm infants. It includes both categorical and continuous variables. The categorical variables represent the occurrence of various conditions and interventions, while the continuous variables represent scores or measurements taken at different time points. The dataset structure is capturing a wide range of variables that influence the health trajectories of neonates and relevant maternal factors. It includes data on pregnancy complications, birth conditions, hospitalization details, and developmental outcomes assessed using the Bayley Scales. The data are collected to ensure an understanding of the neonates' health trajectories and relevant maternal factors, facilitating a detailed analysis of the risk factors affecting neurodevelopmental outcomes in preterm infants [Table 1]. It should be noted that the term "NULL" appears frequently in the dataset. In this context, "NULL" signifies missing or unrecorded data for a particular variable.

**Table 1:** variables in dataset.

Variable Name	Description	Measurement Level	Values/Labels
number	Code number of participants	Scale	-
Sex	Gender of the neonates	Nominal	1.00 = male, 2.00 = female
DM	Diabetes Mellitus in pregnancy	Nominal	1.00 = yes, 2.00 = no
preeclampsia	Occurrence of preeclampsia during pregnancy	Nominal	1.00 = yes, 2.00 = no
hypothyroid	Occurrence of hypothyroidism during pregnancy	Nominal	1.00 = yes, 2.00 = no
PROM	Premature Rupture of Membranes	Nominal	1.00 = yes, 2.00 = no
IUGR	Intrauterine Growth Restriction	Nominal	1.00 = YES, 2.00 = NO
pregnancycomplication	General complications during pregnancy	Nominal	1.00 = yes, 2.00 = no
pneumothorax	Occurrence of pneumothorax during hospitalization	Nominal	1.00 = yes, 2.00 = no
NEC	Occurrence of Necrotizing Enterocolitis during hospitalization	Nominal	1.00 = yes, 2.00 = no
sepsis	Occurrence of sepsis during hospitalization	Nominal	1.00 = yes, 2.00 = no
PDA	Occurrence of Patent Ductus Arteriosus during hospitalization	Nominal	1.00 = yes, 2.00 = no
icter	Occurrence of jaundice during hospitalization	Nominal	1.00 = yes, 2.00 = no
meningitis	Occurrence of meningitis during hospitalization	Nominal	1.00 = yes, 2.00 = no
IVH	Occurrence of Intraventricular Hemorrhage during hospitalization	Nominal	1.00 = yes, 2.00 = no
seizure	Occurrence of seizures during hospitalization	Nominal	1.00 = yes, 2.00 = no
BPD	Occurrence of Bronchopulmonary Dysplasia during hospitalization	Nominal	1.00 = yes, 2.00 = no
B.C	Positive blood culture during hospitalization	Nominal	1.00 = positive, 2.00 = negative
drug.mother	Mother's drug use during pregnancy	Nominal	1.00 = yes, 2.00 = no
laborType	Type of labor (e.g., cesarean)	Nominal	1.00 = NVD, 2.00 = cs
type.of.ressucitation	Gestational age at birth	Nominal	1.00 = ppv, 2.00 = advanced
ehya.badve.tavallod	Necessity for post-birth resuscitation	Nominal	1.00 = yes, 2.00 = NO
surfactant	Administration of surfactant during hospitalization	Nominal	1.00 = yes, 2.00 = no
aggressive.ventilation		Nominal	1.00 = yes, 2.00 = no
notaggressive.ventilation	Duration of hospitalization in days	Nominal	1.00 = yes, 2.00 = no
csf.culture	CSF culture during hospitalization	Nominal	1.00 = Positive, 2.00 = Negatiev
SepsisnegativeCulture	Instances of negative culture during hospitalization	Nominal	1.00 = yes, 2.00 = no
mother.sonogarphy.result	Mother's abnormal ultrasound findings during pregnancy	Nominal	1.00 = normal, 2.00 = abnormal
CognitiveDomain	Birth weight in grams	Ordinal	.00 = 0, 1.00 = < −1 sd, 2.00 = < −2 sd, 3.00 = *n*
LanguageDomain	Impairments in the language domain based on Bayley Scales	Ordinal	.00 = 0, 1.00 = < −1 sd, 2.00 = < −2 sd, 3.00 = *n*
PerceptualDomain	Impairments in the perceptual domain based on Bayley Scales	Ordinal	.00 = 0, 1.00 = < −1 sd, 2.00 = < −2 sd, 3.00 = *n*
finemotor	Impairments in the fine motor domain based on Bayley Scales	Ordinal	.00 = 0, 1.00 = < −1 sd, 2.00 = < −2 sd, 3.00 = *n*
coarsemotor	Impairments in the coarse motor domain based on Bayley Scales	Ordinal	.00 = 0, 1.00 = < −1 sd, 2.00 = < −2 sd, 3.00 = *n*
correctedage	Corrected age in days	Scale	1 = < −1 sd, 2 = < −2 sd, 3 = Normal
Age	Current age in days	Scale	1 = < −1 sd, 2 = < −2 sd, 3 = Normal
lang.recode	Impairments in the language domain based on Bayley Scales	Ordinal	1.00 = < −2 sd, 2.00 = < −1 sd, 3.00 = Normal
percep.recode	Impairments in the perceptual domain based on Bayley Scales	Ordinal	1.00 = < −2 sd, 2.00 = < −1 sd, 3.00 = Normal
fine.recode	Impairments in the fine motor domain based on Bayley Scales	Ordinal	1.00 = < −2 sd, 2.00 = < −1 sd, 3.00 = Normal
coarse.recode	Impairments in the coarse motor domain based on Bayley Scales	Ordinal	1.00 = < −2 sd, 2.00 = < −1 sd, 3.00 = Normal
cog.recode	Impairments in the cognitive domain based on Bayley Scales	Ordinal	1.00 = < −2 sd, 2.00 = < −1 sd, 3.00 = Normal
lang.cat	Impairments in the language domain based on Bayley Scales	Nominal	.00 = abnormal, 1.00 = normal
percep.cat	Impairments in the perceptual domain based on Bayley Scales	Nominal	.00 = abnormal, 1.00 = normal
fine.cat	Impairments in the fine motor domain based on Bayley Scales	Nominal	.00 = abnormal, 1.00 = normal
cog.cat	Impairments in the cognitive domain based on Bayley Scales	Nominal	.00 = abnormal, 1.00 = normal
coarse.cat	Impairments in the coarse motor domain based on Bayley Scales	Nominal	.00 = abnormal, 1.00 = normal

## 4 Experimental Design, Materials and Methods

### 4.1 Study framework

This research was structured as a retrospective cohort study, designed to scrutinize the myriad of neonatal and maternal factors that potentially influence the developmental outcomes of preterm infants [2].

Participant Cohort

The study population included 89 preterm infants initially admitted to the NICU of Ghaem Hospital, Mashhad, between December 2016 and January 2020. These subjects were later re-evaluated to assess their developmental status.

Inclusion Criteria

Age greater than 2 months at the time of developmental assessment.

Complete medical records available for review.

Availability of Bayley Scales of Infant and Toddler Development scores.

Exclusion Criteria

Incomplete medical records.

Presence of severe congenital anomalies affecting neurodevelopment.

Data collection was a multi-step process, focusing on different facets of neonatal and maternal health:

### 4.2 Methodology of data acquisition

The data acquisition process was orchestrated in several phases, each focusing on different aspects of neonatal and maternal health:

Neonatal Data: This phase involved the collection of data pertaining to the neonates' gender, chronological age, and corrected age, a metric fine-tuned based on the gestational age at birth.

Maternal Data: This segment was dedicated to the exhaustive documentation of maternal aspects, encompassing a spectrum of complications encountered during pregnancy, inclusive of Diabetes Mellitus, preeclampsia, and hypothyroidism. Additionally, data on maternal drug usage and abnormal ultrasound findings during the gestational period were collated.

Birth Metrics: This segment focused on gathering data related to the neonates' birth conditions, encapsulating metrics such as birth weight, head circumference at birth, and the nature of labor, supplemented by Apgar scores documented at one and five minutes post-birth.

Hospitalization Chronicles: This phase chronicled an array of data points during the neonates' hospitalization period, encompassing medical interventions and complications encountered, thereby forming a rich repository of data for analysis.

### 4.3 Analytical framework

The analytical framework adopted for this study was grounded in the utilization of descriptive statistics, non-parametric tests, and binary logistic regression methodologies. Leveraging the capabilities of SPSS V.26 and R programming software, an analysis was undertaken to decipher patterns and correlations within the data.

### 4.4 Instrumentation

Central to the evaluation of neurodevelopmental outcomes is the utilization of the Bayley Scales of Infant and Toddler Development, a prominent instrument in pediatric assessment. This tool facilitates a granular analysis across a spectrum of developmental domains.

The Bayley Test stands as a pivotal tool extensively employed in scrutinizing the developmental trajectory in infants and toddlers [3]. It encompasses a broad spectrum of developmental aspects, including cognitive, motor, and linguistic capabilities, thereby establishing itself as a cornerstone in exhaustive developmental research. The test is structured around a series of tasks that scrutinize a child's motor proficiencies and cognitive abilities, alongside their comprehension and articulation of language. This deep-dive analysis furnished by the Bayley Test furnishes a panorama of a child's developmental stage, pinpointing potential areas of delay or concern. Consequently, it serves as a catalyst in formulating intervention strategies, amplifying the efficacy of early intervention services for children who necessitate additional developmental support [4], [5], [6].

Furthermore, to offer a well-rounded perspective on the developmental milestones achieved by the subjects, results were delineated using two distinct parameters. Initially, each domain of the Bayley scale underwent an analysis to ascertain the Z-score, providing a statistical insight into a score's relation to the group's mean score (7).

Moreover, these Z-scores were subsequently categorized into ``normal'' and ``abnormal'' classifications to foster a clearer understanding of the developmental statuses. A Z-score of −1 or below in any specific domain was identified as a marker of potential developmental delays of varying degrees, labeled as ``abnormal''. In contrast, scores exceeding −1 were denoted as "normal", signifying a regular developmental pathway. This bifurcated parameter approach not only facilitates a statistical evaluation but also offers a clinically pertinent interpretation of the developmental advancements in the neonates, thereby enriching the depth and practicality of the research outcomes.

### 4.5 Software utilization

Statistical analyses were performed using the R studio (version 3.5.3, R Core Team, 2019) and SPSS version 26 (SPSS Inc., Chicago, Illinois, USA).

### 4.6 Sample size determination

The initial phase of the study was orchestrated within the confines of the NICU at Ghaem hospital, Mashhad, characterized by a controlled environment conducive to neonatal care.

Using the data obtained from the study conducted by Sung Ho Ahn (2), where the prevalence of cognitive, language, and motor delays in preterm children was found to be 38%, 26%, and 35% respectively, the sample size was calculated considering an alpha of 0.05 and *d* = 0.3p for these outcomes, resulting in respective sample sizes of 70, 122, and 80. The calculations were as follows: Alpha (α): 0.05, Critical value (Z1-α/2): 1.96, Margin of error (d): 0.078, Proportion (p): 0.26, Sample size (n): 122

## Limitations

The primary limitation encountered during this study was the extensive number of variables that were scrutinized, which necessitated a substantial allocation of resources, both in terms of budget and personnel. This, unfortunately, restricted us from expanding the sample size to a more extensive cohort, which could have potentially offered a more insight into the developmental trajectories of preterm infants.

## Ethics Statement

This research was conducted in strict adherence to the principles outlined in the Declaration of Helsinki. All procedures involving human participants were reviewed and approved by the Ethics Committee of Mashhad University of Medical Sciences. The research protocol was duly registered under protocol number IR.MUMS.MEDICAL.REC.1398.590. Furthermore, we affirm that informed consent was obtained from all subjects legal guardians, ensuring the voluntary participation of individuals in this study.

## CRediT authorship contribution statement

**Azadeh Darabi:** Conceptualization, Methodology, Writing – original draft, Project administration, Supervision. **Raheleh Faramarzi:** Data curation, Formal analysis, Investigation, Validation, Writing – review & editing. **Hassan Boskabadi:** . **Gholamali Maamouri:** . **Reyhane Rezvani:** Resources, Software, Visualization, Funding acquisition, Investigation.

## Data Availability

development preterm infant (Original data) (Mendeley Data)
